# A New Xanthone from *Moutabea guianensis* Aubl

**DOI:** 10.3390/molecules19078885

**Published:** 2014-06-26

**Authors:** Haroldo da S. Ripardo Filho, Luidi C. Pacheco, Edinaldo da S. Andrade, Marivaldo José C. Correa, Gisele Maria S. P. Guilhon, Lourivaldo S. Santos

**Affiliations:** Instituto de Ciências Exatas e Naturais, Universidade Federal do Pará-UFPA, Rua Augusto Corrêa, S/N, 66075-900 Belém-PA, Brazil; E-Mails: ripardofilho@ufpa.br (H.S.R.F.); luidicp@hotmail.com (L.C.P.); edinaldo.metalurgico@gmail.com (E.S.A.); majocost@ufpa.br (M.J.C.C.); Giselle@ufpa.br (G.M.S.P.G.)

**Keywords:** *Moutabea guianensis*, Polygalaceae, xanthone, structural elucidation, spectroscopic analysis

## Abstract

The ethyl acetate extract of the roots of *Moutabea guianensis* gave 1,6-dihydroxy-4,7,8-trimethoxy-9*H*-xanthen-9-one (**1**), a new xanthone. The isolation was accomplished by column chromatography on silica gel and the structural elucidation of this compound was established by spectroscopic analyses including 1D and 2D NMR and HRESIMS.

## 1. Introduction

Polygalaceae plants have been the source of many xanthones [1,2], in addition to coumarins, phenols, triterpenes, steroids, pyrones derivatives and alkaloids [3,4,5]. These species contain chemical compounds with a large spectrum of biological activities [6], including anti-depressant [7] and anti-angiogenic [8]. *Moutabea guianensis* Aubl is a Polygalaceae plant of the Amazon area and recently, we carried out a chemical and phytotoxic investigation with its roots. The chemical investigation afforded three known steroids spinasterol, spinasterone and glucopyranosylspinasterol [9].

**Figure 1:**
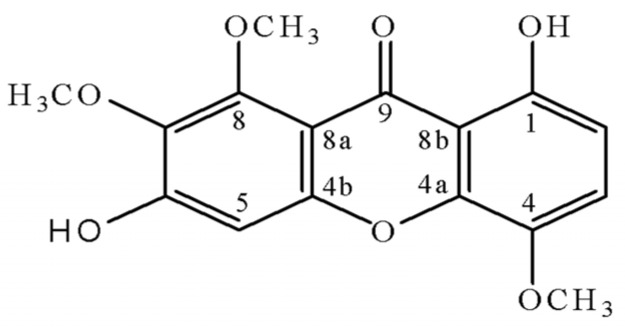
Structure of compound **1**.

As a part of ongoing research to characterize the chemical components in the roots of *M. guianensis*, we now report the isolation and structural elucidation of a new xanthone. It was identified as 1,6-dihydroxy-4,7,8-trimethoxy-9*H*-xanthen-9-one (**1**), and named moutabeone (Figure 1).

## 2. Results and Discussion

The ^1^H-NMR spectrum of compound **1** showed eight signals, three singlets between δ 3.94–4.03 indicative of three methoxyl groups; three aromatic hydrogens at δ 6.68 and 7.18 (two doublets with *J* = 9.0 Hz) and δ 6.90 (*s*) and one singlet at δ 12.52 assigned to one chelated OH group placed at C_1_. The ^13^C-NMR spectrum further reveals signals of three methoxyl carbons (δ 57.5–61.9), three methine carbons (δ 99.3, 108.9, 119.8), ten non hydrogenated carbons, including one carbonyl group at δ 181.3 characteristic for a monochelated carbonyl carbon [10], two which have no oxygen substituent (both at δ 109.8) and seven of which have an oxygen substituent (137.6–155.9). The unambiguous attribution was established by means of two-dimensional NMR spectroscopic techniques. The chemical assignments of methine carbons C_2_, C_3_, C_4_ and methoxyl carbons in the ^13^C-NMR were achieved by HETCOR experiment [^1^*J*(CH)]. The other chemical shifts were assigned by long-range [^2^*J*(CH), ^3^*J*(CH) and ^4^*J*(CH)] correlation observed in the HMBC spectrum (Table 1). Its molecular formula (C_16_H_14_O_7_) was determined based on a peak in the HRESIMS data at *m/z* 319.0817 [M+H]^+^ (Calculated for C_16_H_15_O_7_), suggesting 10 degrees of unsaturation. These data were sufficient to consider the possibility of a pentasubstituted xanthone structure with two hydroxyl groups at C_1_ and C_6_. Thus, the structure of **1** was fully elucidated, and it was named moutabeone.

**Table 1:** ^1^H-NMR (300 MHz) and ^13^C-NMR (75 MHz) spectral data for compound **1** in CDCl_3_.

Positions	δH ( *J* in Hz)	δC	DEPT	HMBC (H→C)	
1	-	154.9	C		
2	6.68 (d, 9.0)	108.9	CH	8b, 1, 4	
3	7.18 (d, 9.0)	119.8	CH	1, 4a, 4	
4	-	139.4	C		
4a	-	145.1	C		
4b	-	154.5	C		
5	6.90 (s)	99.3	CH	8a, 9, 4b, 6, 7	
6	-	155.9	C		
7	-	137.6	C		
8	-	152.2	C		
8a	-	109.3	C		
8b	-	109.3	C		
9	-	181.3	C	
4-OCH_3_	3.93 (s)	57.5	CH_3_	4
7-OCH_3_	4.02 (s)	61.7	CH_3_	7
8-OCH_3_	4.00 (s)	61.9	CH_3_	8
1-OH	12.52 (s)	-	-	2, 1, 8b

## 3. Experimental

### 3.1. General Information

NMR spectra were recorded on a Varian 300 MHz NMR spectrometer (300 MHz and 75 MHz for ^1^H and ^13^C, respectively) using TMS as internal standard. HRESIMS was carried out on a Waters Xevo G2-S QTof/Tof spectrometer. IR was carried out on a Shimadzu Prestige 21. Column chromatography was performed on silica gel (70–230 mesh, MACHEREY-NAGEL; Düren, Germany). TLC was performed on silica gel 60 F254 (Sorbent Technologies, Sorbent Technologies; Norcross, GA, USA) plates. Spots were visualized by spraying with aqueous H_2_SO_4_ (15%) saturated with CeSO_4_ solution, followed by heating.

### 3.2. Plant Material

The roots of *M. guianensis* were collected in the experimental field of Embrapa Amazônia Oriental, located in Belém, Pará State, Brazil, on March 2012. A voucher specimen (195862) was kept in the Herbarium MG of the Museu Paraense Emílio Goeldi (MPEG). Roots were dried on forced air circulation on 40 °C for five days and powdered in a knife mill.

### 3.3. Extraction and Isolation

The roots of *M. guianensis* (928 g) dried and powdered, were submitted to successive extractions with hexane (3 L), ethyl acetate (3 L) and methanol (3 L) at room temperature for five days. After removal of the solvent *in vacuo*, the hexane, ethyl acetate and methanol extracts were obtained, respectively. The ethyl acetate extract (2.0 g) was subjected to silica gel column chromatography and eluted with hexane-EtOAc and EtOAc-MeOH, collecting 20 fractions of 125 mL each. The fractions were combined according to TLC to give seven groups (G_1_–G_7_). G_4_ (254.5 mg) was rechromatographed on silica gel with hexane-EtOAc as eluent, collecting 170 fractions of 13 mL each. The fractions 111–117 were combined according to TLC to afford compound **1** (32 mg) as a yellow amourphous solid. Yellow amorphous solid; ^1^H-NMR and ^13^C-NMR data see Table 1; UV λ_max_/nm (acetonitrile-water): 199, 240, 284, 314, 358 (*sh*). IR (KBr) 3475, 2999, 2939, 2837, 1602, 1485, 1471, 1288, 1228, 1099, 1008, 981, 948, 794, 736, 609 cm^−1^. HRESIMS *m/z*: 319.0817 [M+H]^+^ (calcd for C_16_H_15_O_7_).

## 4. Conclusions

A new xanthone 1,6-dihydroxy-4,7,8-trimethoxy-9*H*-xanthen-9-one (**1**), was isolated from the ethyl acetate extract of the roots of *M. guianensis*. This compound is expected to be an antioxidant and free radical scavenger like other xanthones and its biological activity will be studied in future.

## Supplementary Materials

Supplementary materials can be accessed at: http://www.mdpi.com/1420-3049/19/7/8885/s1.
