# Supplementary material for: A New Xanthone from Moutabea guianensis Aubl
Source: Molecules. 2014 Jun 26;19(7):8885–9. doi: 10.3390/molecules19078885 (PMC6271822; doi:10.3390/molecules19078885)

Supporting Information

Figure S1. ^{1}H-NMR (300 MHz, $CDCl_3$) spectrum of compound **1**.

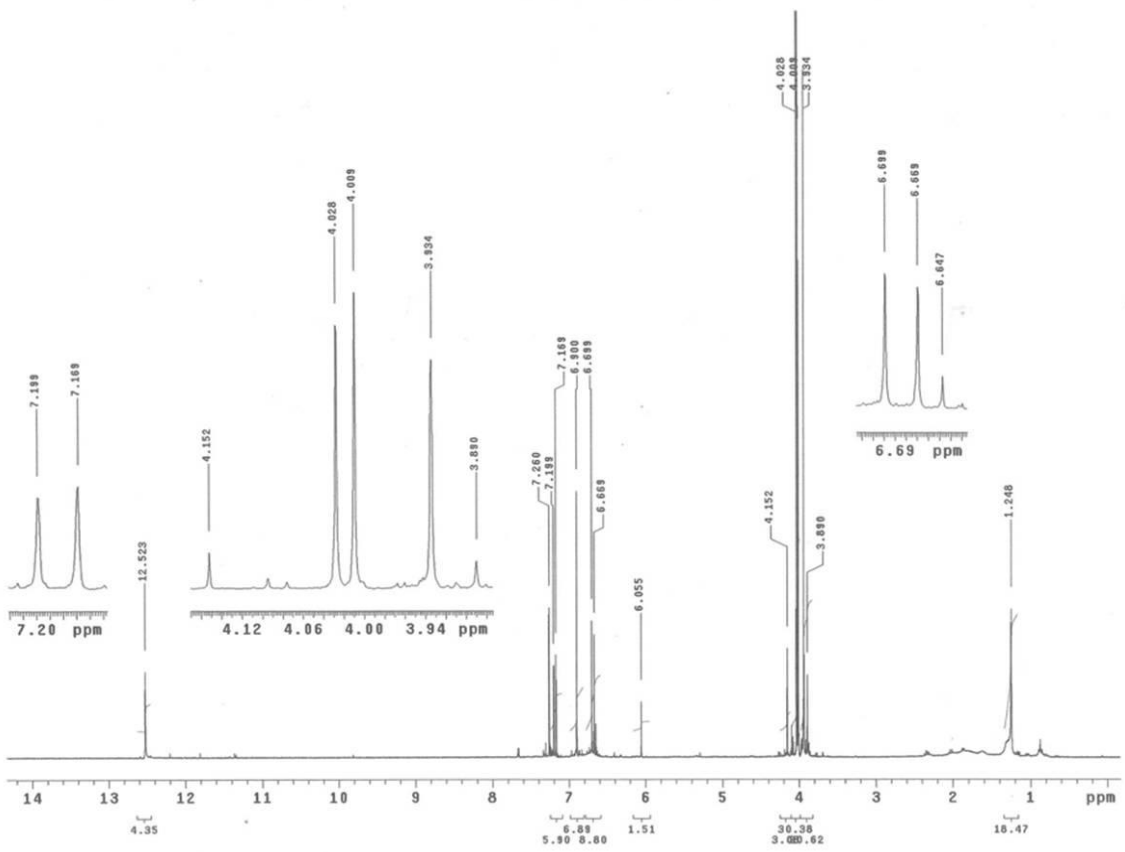

Figure S2. ^{13}C-NMR (75 MHz, $CDCl_3$) spectrum of compound **1**.

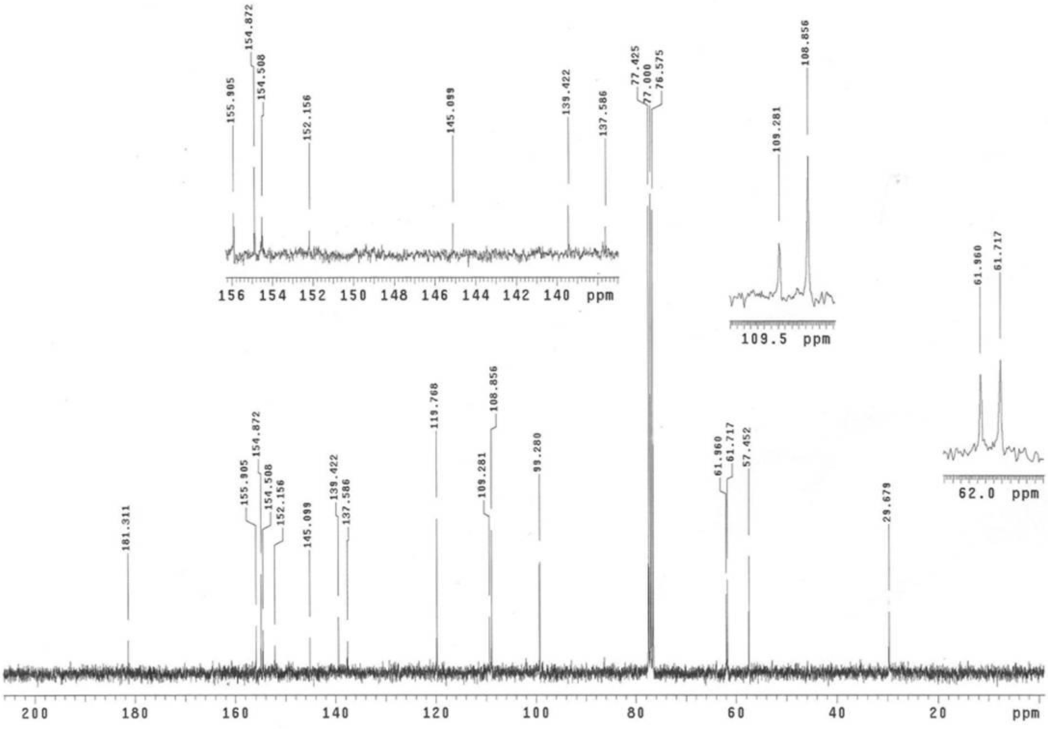

Figure S3. DEPT spectrum of compound **1**.

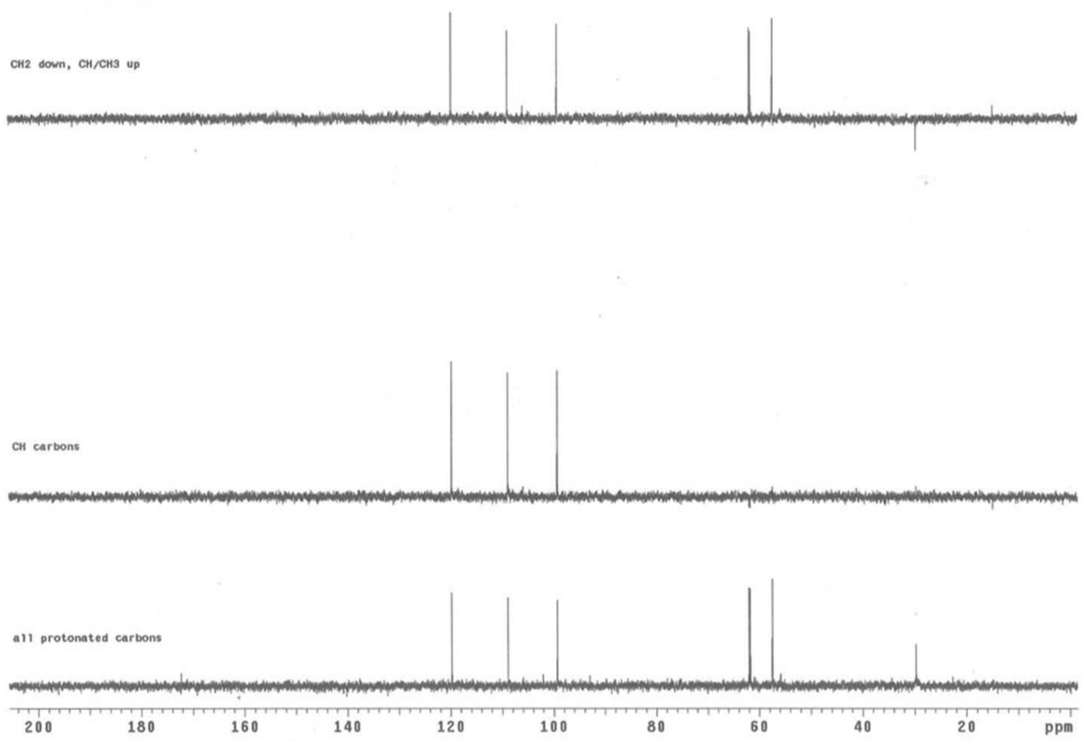

Figure S4. COSY spectrum of compound **1**.

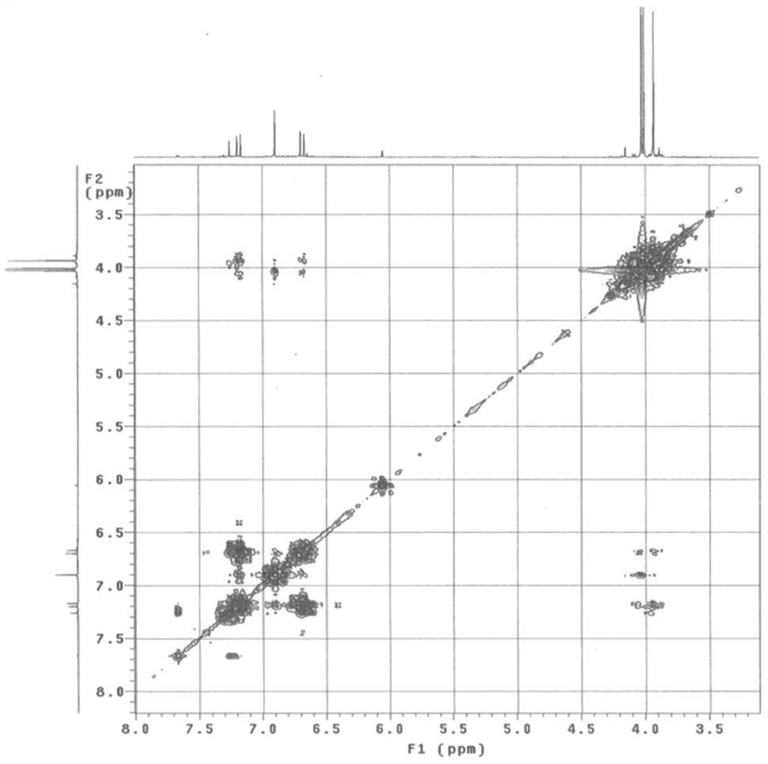

Figure S5. HETCOR spectrum of compound **1**.

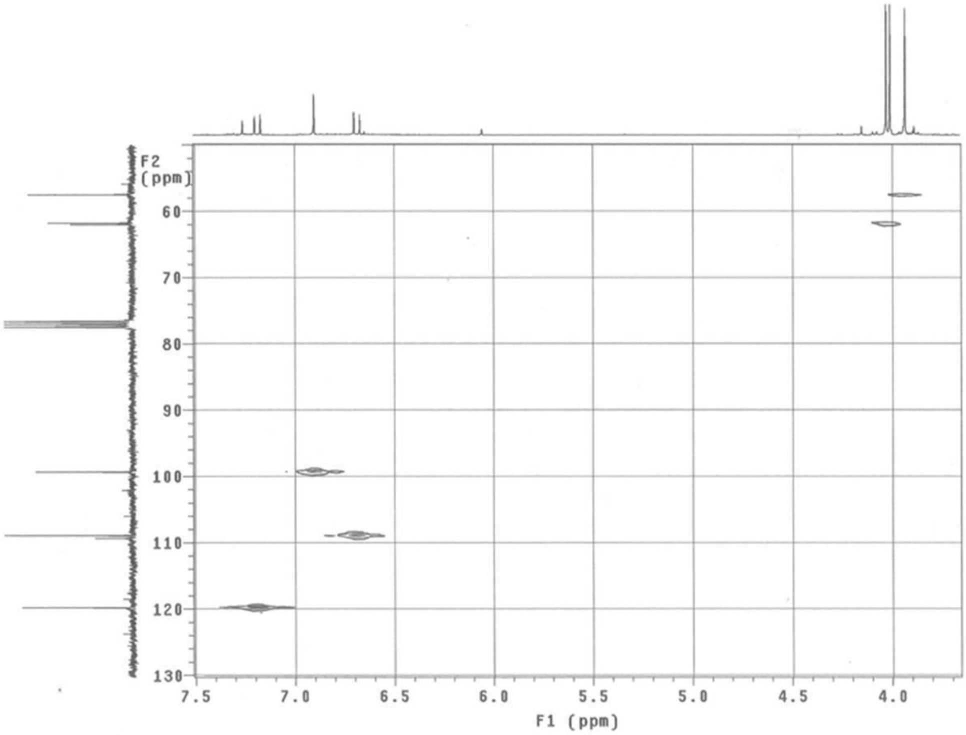

Figure S6. HMBC spectrum of compound **1**.

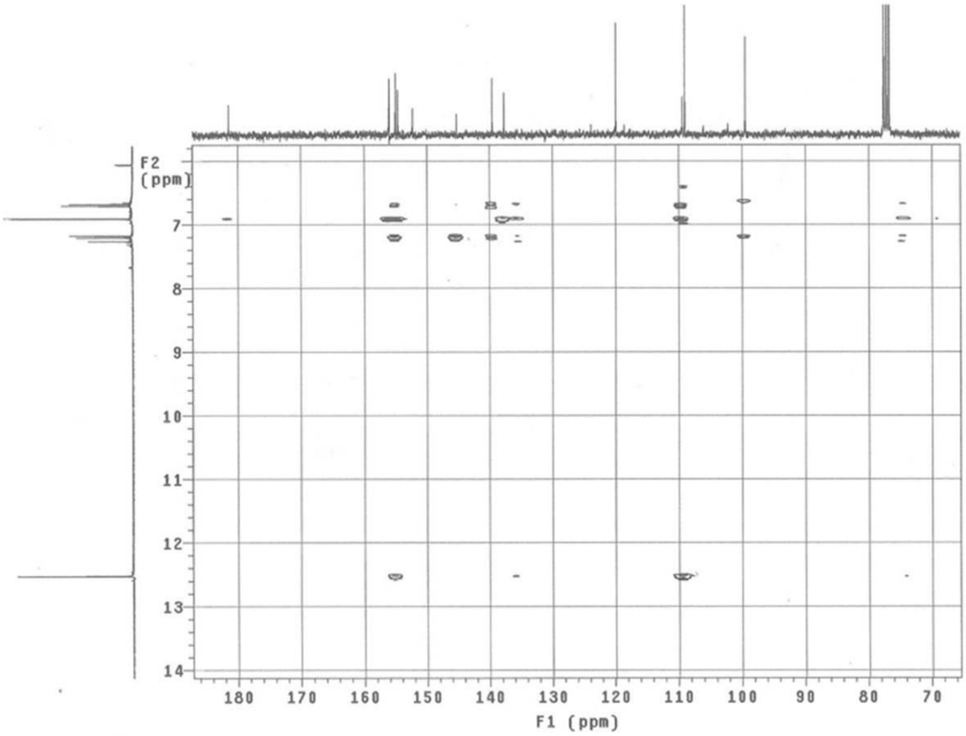

Figure S7. Expansion of HMBC spectrum of compound **1**.

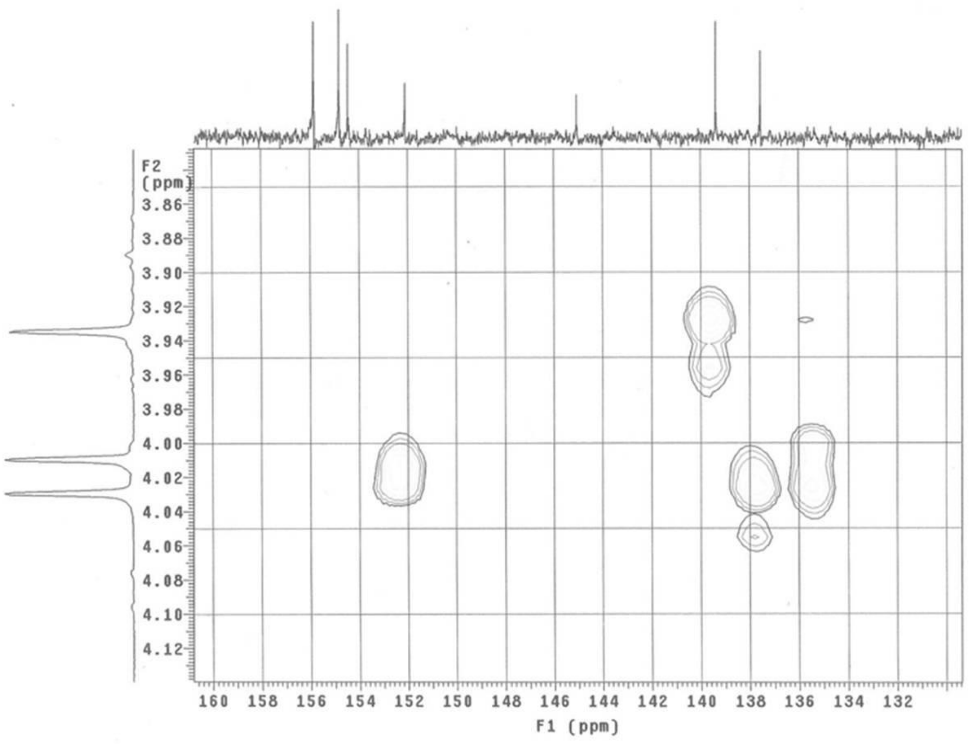

Figure S8. HRESIMS spectrum of compound **1**.

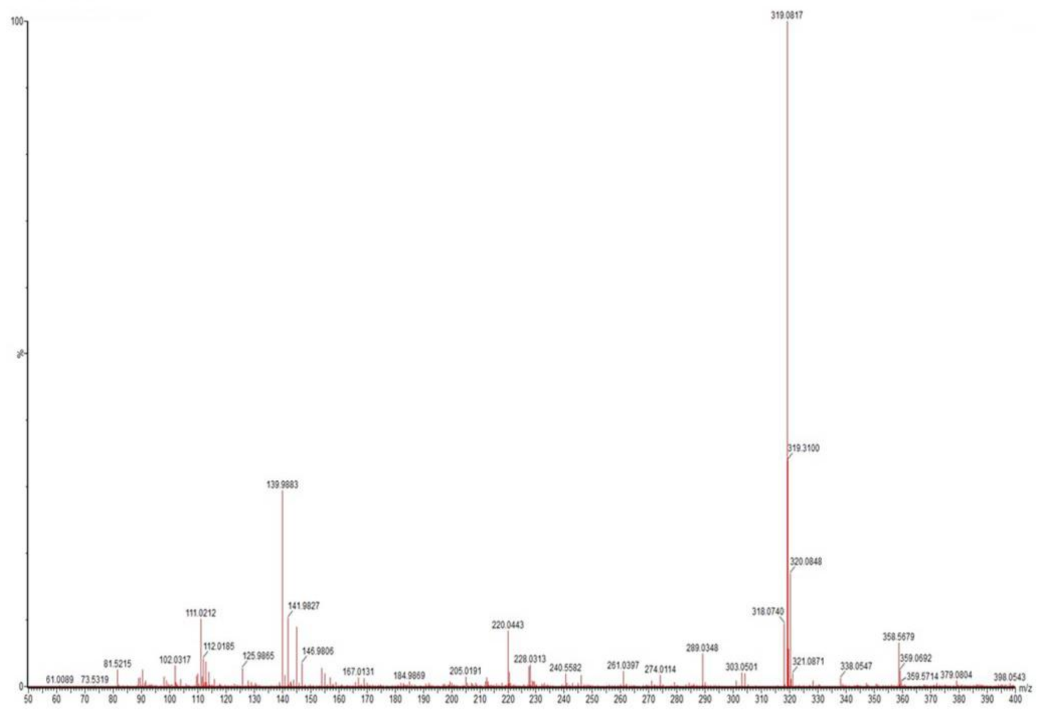

Figure S9. UV spectrum of compound **1**.

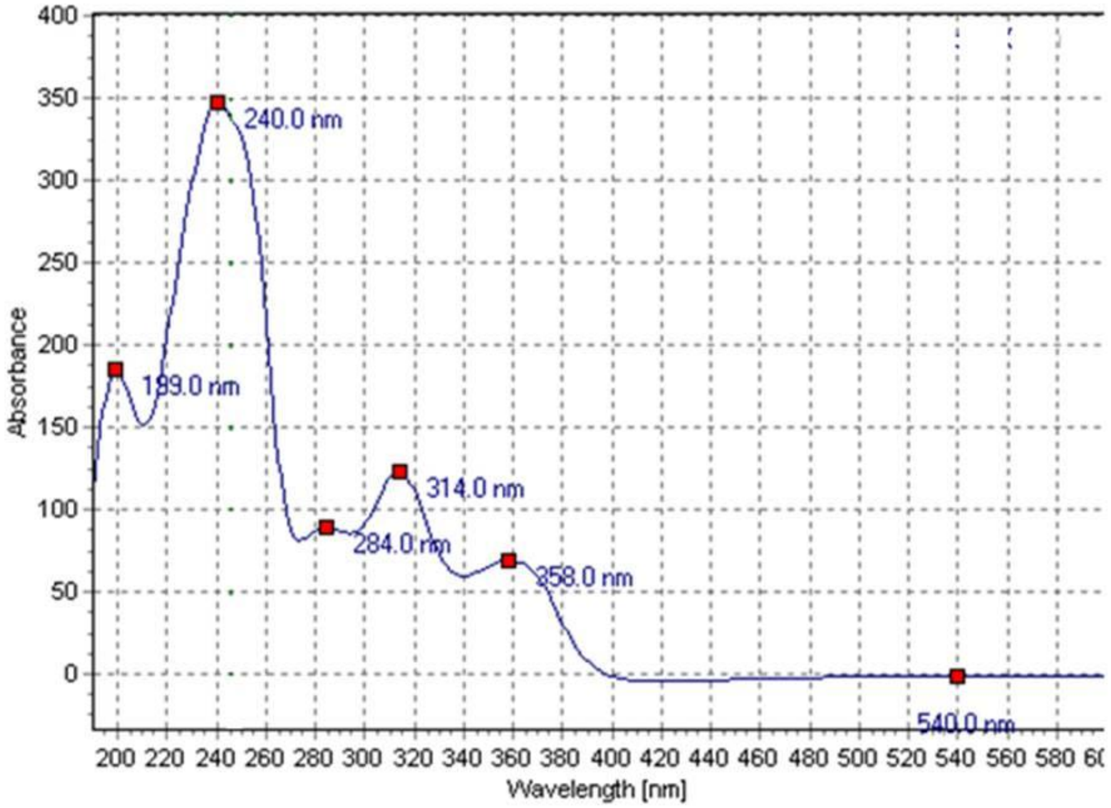

Figure S10. IR spectrum of compound **1**.

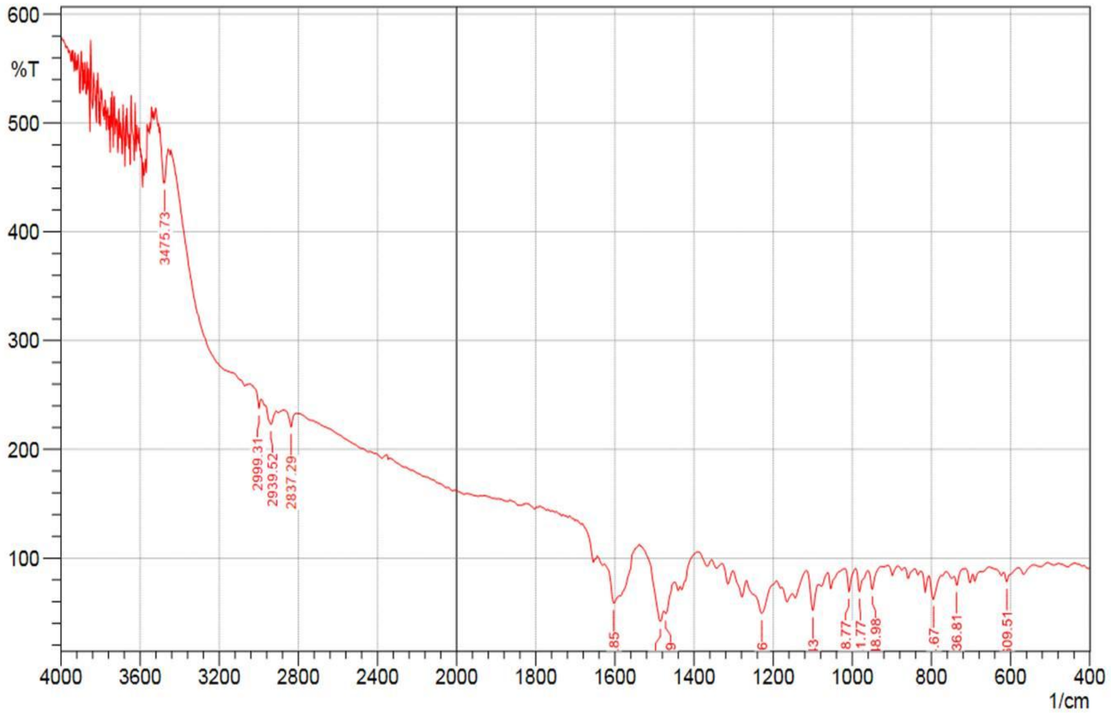

Supplement: Supplementary file 1 [file molecules-19-08885-s001.pdf]
